# Dynamic Macrophages: Understanding Mechanisms of Activation as Guide to Therapy for Atherosclerotic Vascular Disease

**DOI:** 10.3389/fcvm.2018.00097

**Published:** 2018-08-03

**Authors:** Julius L. Decano, Masanori Aikawa

**Affiliations:** ^1^Center for Interdisciplinary Cardiovascular Sciences, Division of Cardiovascular Medicine, Department of Medicine, Brigham Women's Hospital, Harvard Medical School, Boston, MA, United States; ^2^Center for Excellence in Vascular Biology, Division of Cardiovascular Medicine, Department of Medicine, Brigham Women's Hospital, Harvard Medical School, Boston, MA, United States; ^3^Channing Division of Network Medicine, Department of Medicine, Brigham Women's Hospital, Harvard Medical School, Boston, MA, United States

**Keywords:** macrophages, cardiovascular diseases, drug development, inflammation, cell metabolism

## Abstract

An emerging theory is that macrophages are heterogenous; an attribute that allows them to change behavior and execute specific functions in disease processes. This review aims to describe the current understanding on factors that govern their phenotypic changes, and provide insights for intervention beyond managing classical risk factors. Evidence suggests that metabolic reprogramming of macrophages triggers either a pro-inflammatory, anti-inflammatory or pro-resolving behavior. Dynamic changes in bioenergetics, metabolome or influence from bioactive lipids may promote resolution or aggravation of inflammation. Direct cell-to-cell interactions with other immune cells can also influence macrophage activation. Both paracrine signaling and intercellular molecular interactions either co-stimulate or co-inhibit activation of macrophages as well as their paired immune cell collaborator. More pathways of activation can even be uncovered by inspecting macrophages in the single cell level, since differential expression in key gene regulators can be screened in higher resolution compared to conventional averaged gene expression readouts. All these emerging macrophage activation mechanisms may be further explored and consolidated by using approaches in network biology. Integrating these insights can unravel novel and safer drug targets through better understanding of the pro-inflammatory activation circuitry.

## Introduction

In the last few decades, accumulating evidence has supported modulation of inflammatory signals, and regulation of immune cell to cell interactions in atherosclerosis are key therapeutic strategies for atherothrombotic disease (1–3) The recent Canakinumab Antiinflammatory Thrombosis Outcome Study (CANTOS) trial, involving over 10,000 patients, show conclusive proof that reduction of inflammation, specifically targeting the interleukin-1β (IL-1β) pathway activation, independent of LDL cholesterol lowering, can significantly lower coronary artery disease (CAD) morbidity and mortality. Antagonizing the IL-1β signaling resulted in marked reduction of plasma high-sensitivity C-reactive protein (hs-CRP) levels among patients with elevated hs-CRP levels and history of myocardial infarction, which eventually led to decreased major adverse cardiac/CV events (MACE and MACE+) (4) hs-CRP is a predictive marker of the severity of atherosclerosis and extent of future cardiovascular events (5, 6). Success of anti-inflammatory drug trials are reliant on rigorous basic science research, integrating a plurality of approaches. These include analyses of pathologic specimens, tightly controlled *in vitro* experiments and extensive use of pre-clinical small animal models to gather basic mechanistic information about the disease. In the context of chronic inflammation in cardiovascular disease, basic science research in macrophage biology has undoubtedly been the guiding compass for pursuing this anti-inflammation focus of atherosclerosis therapy.

## Macrophage activation in vascular inflammatory disease

Both acute and chronic forms of vascular inflammation are typified by the multitude of vasculitides and atherothrombotic pathologies. CAD, peripheral artery disease, vein graft failure, and arterio-venous fistula failure have seen various macrophage subtypes playing crucial roles. They either drive disease progression or cessation, or promote vessel repair and healing (7). Understanding the various phenotypes that allow macrophages to be categorized into subclasses with stereotyped behavior and function is crucial. This helps design strategies to precisely modulate immune signaling in vascular inflammation (1–3, 8). By limiting cellular subpopulations promoting plaque development, intimal cell proliferation, and tissue damage may be mitigated. This may also spare the subpopulation deemed beneficial for achieving disease control and resolution to allow return to homeostasis. In addition, understanding the profound adaptability, and plasticity of macrophages is key to knowing how to trigger phenotypic and functional changes within these cells and how far they can be reprogrammed.

What we have learned from the past is that both *in vitro* modeling of human and mouse primary macrophages complemented by experiments on small animal models of vascular disease have been important in elucidating mechanisms of macrophage activation and their role in the progression of the atherothrombotic lesions in CAD (9, 10). It is known that majority of the release of matrix metalloproteinases, MMPs, in human atherosclerotic plaques may derive from macrophages and foam cells, and to a lesser extent from smooth muscle cells (SMCs) and endothelial cells (ECs) (11). Excessive activation of proteases in the lesion lead to increased degradation of fibrillar collagen. This determines plaque integrity, leading to friable and unstable lesions. This may also lead to adverse remodeling prompting rupture and embolism of plaque debris, often seen in plaques (12, 13). Our preclinical studies used genetically-altered mouse strains to demonstrate that MMP-collagenases, major macrophage products, indeed promote the paucity of plaque collagen (14, 15). In vein grafts, MMP-2 and MMP-9 may play important roles in degrading the basement membrane which leads to enhanced infiltration of pro-inflammatory monocyte and macrophage populations (16, 17). These unstable plaque features are also most prominent among CAD patients with elevated low density lipoprotein (LDL) cholesterol levels (18), elevated lipoprotein (a) [Lp(a)] (19) and other metabolic derangements, reiterating a close interplay between inflammation and dysregulation of lipid handling (and other metabolic syndromes). LDL modifications, LDL cholesterol efflux and reverse cholesterol efflux all contribute to how cholesterol crystals instigate initial stages of atherosclerosis.

The process begins with recruiting monocytes from the circulating blood, followed by several processes including their differentiation into macrophages, foam cell formation and activation of the NOD-like receptor-pyrin domain (PYD)- containing-3 (NLRP3) inflammasome complex. Cholesterol crystals may cause phagolysosomal damage in macrophages priming them to activate NLRP3. Activation of NF-κB induces macrophages to produce pro-IL-1β and a pro-form of NLRP3. Upon activation of NLRP3, activated caspase-1 cleaves the pro-IL-1β releasing IL-1β, which amplifies the cascade of inflammatory signals (20, 21) including IL-6, tumor necrosis alpha (TNF-α) and pro-thrombotic initiators such as tissue factor (coagulation factor III). Clinical relevance for this is reflected in patients with typically high serum LDL cholesterol levels having increased incidence and severity of CAD and subsequent MACE sequelae. This paved way for the extensive use of statins in aggressively lowering elevated LDL cholesterol levels. Eventually, aggressive lipid lowering therapy indeed improved survival rates, as well as MACE/MACE+ outcomes as shown in clinical studies (22–25). However, certain patient populations did not benefit much due to having a baseline coronary atheroma predominantly dictating their prognosis (MACE) (26, 27). This leads to the burgeoning field of statin related research focused on elucidating mechanisms of statins that mitigate inflammation in cardiovascular disease, independent of their cholesterol lowering action.

For recalcitrant cases, LDL cholesterol levels can be further lowered by increasing availability of hepatic clearance via LDL receptors. Here, reduction of proprotein convertase subtilisin/kexin type 9 (PCSK9) activity and circulating levels have been quite effective, as seen in the success of drugs like evolucumab (Repatha) (28) and alirocumab (Praluent) (21, 29). Whether these inhibiting antibodies can reduce macrophage activation in coronary lesions remains to be proven. Interestingly, with successful reduction of LDL cholesterol levels in the at-risk population, physicians have inevitably selected for and identified a subpopulation of patients with considerable CAD morbidity despite sufficiently lowered LDL cholesterol levels. Since then, multiple points of evidence recognize inflammation, beyond hyperlipidemia, as a key regulatory hub by which CAD risk factors, co-morbid metabolic disease, and cardiovascular adverse events intersect (5, 30).

## Mechanisms for macrophage activation

### Traditional thoughts on macrophage inflammatory pathways in CAD

For years, many thought of resting macrophages as being activated in disease states into polarized subclasses that are diametrically opposed. Either they are “classically-activated” or pro-inflammatory, designated as “M1” and “alternatively-activated” or non/anti-inflammatory/pro-resolving “M2.” However, with much utilization of techniques allowing for multiple parametric assessments like single cell assays (FACS, etc), -OMICs profiling, and networks medicine approach, a more recent multi-dimensional model has emerged (31–35). Unsurprisingly, many authors have shied away from the M1 and M2 designations. Our recent study used single cell gene expression analysis to reveal that interferon gamma (INFγ)-induced “classically-activated” human primary macrophages remain largely heterogeneous, which is not consistent with a traditional “polarization” theory (36).

**Figure 1:**
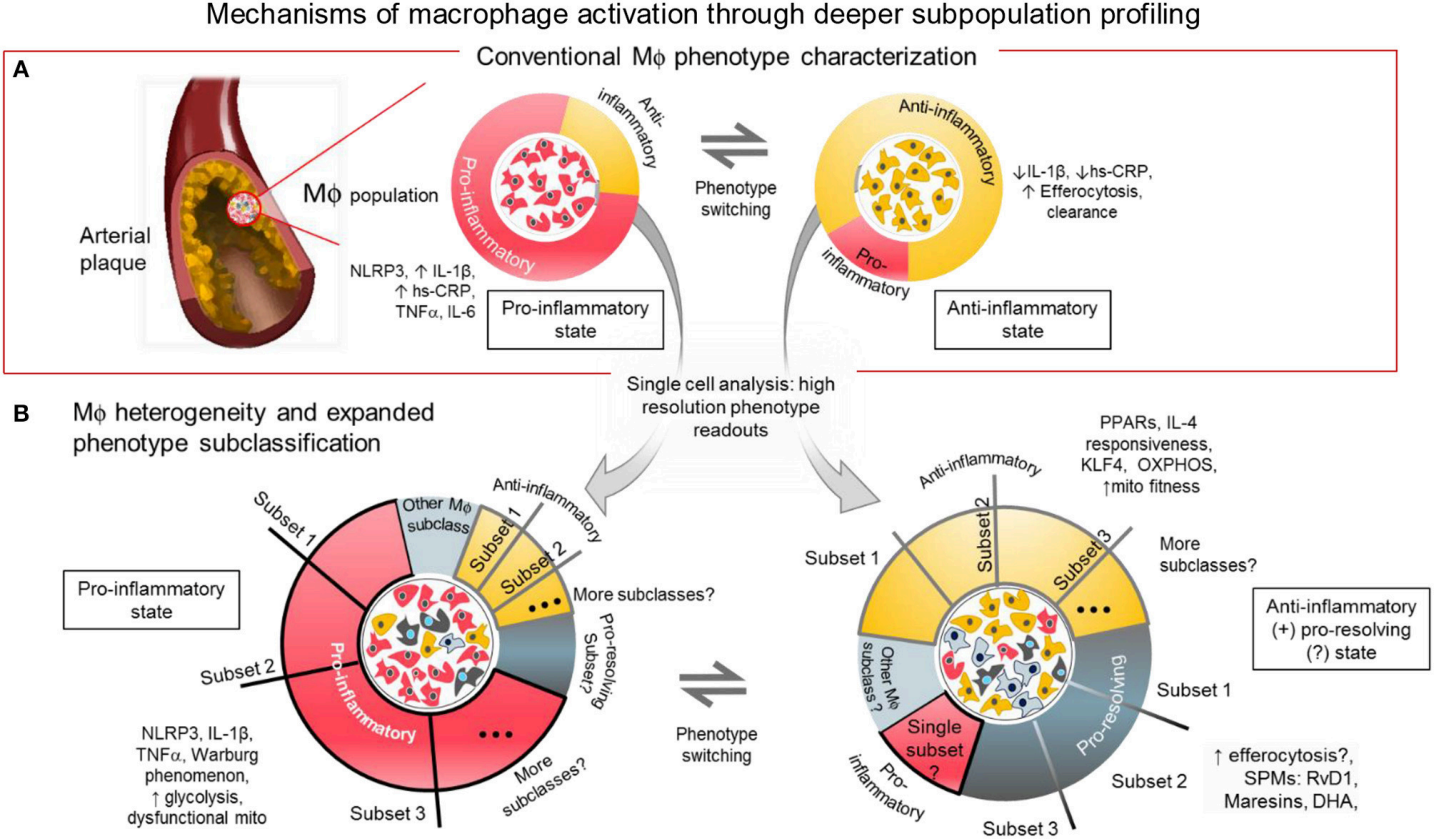
Mechanisms of macrophage activation through deeper subpopulation profiling. **(A)** Conventional characterization of macrophages. Macrophages and foam cells in atherosclerotic lesions comprise a mixture of phenotypes and different activation states. The balance of proinflammatory, and anti-inflammatory/pro-resolving states may determine the fate of the plaque whether it will be stable or prone to rupture. The balance of inflammatory signaling molecules, metabolic states and presence of SPMs, among others, may influence the level of macrophage activation in distinct local regions within the atherosclerotic plaque. **(B)** Macrophage heterogeneity and expanded subclassification. The balance of factors within the macrophages may depend on the number and size of subpopulations with distinct functional phenotypes as revealed by single cell analysis. One or more subset populations within a specifically activated macrophage population and a disproportional balance of any one subset may drive the outcome of disease progression. Identifying the driver population(s) may be the key to identifying regulators of the disease.

Acknowledging that macrophage subclasses fall into a spectrum of activation that is beyond bi-directional is the currently accepted paradigm. However, it may be still helpful, from a drug development and therapeutic research stand point, to think of macrophages in CVD to be either pro-inflammatory state(s) or any state(s) that is otherwise, most likely either immunosuppressive or pro-resolving (2, 37), (Figure 1A) because cause-effect relations are clear in these systems. So-called M1 macrophages are the ones that generate pro-inflammatory cytokines like TNF-α, inducible nitric oxide synthase (iNOS), and IL-6 (38). INFγ or lipopolysaccharide (LPS) is used to promote the pro-inflammatory phenotype *in vitro* (32) by triggering toll-like receptor (TLR)4 signaling associated pathways NF-κB, Notch, and INFγ/STAT-1. The NF-κB and NLRP3 inflammasome pathways are enhanced in pro-inflammatory macrophages. As stated above, experiments in small animal models reveal microscopic cholesterol crystals that activate the NLRP3 inflammasome resulting in the secretion of interleukin 1 family of cytokines (20). In another pathway, Delta-like 4 ligand (Dll4)-triggered Notch signaling can activate the NF-κB pathway to induce pro-inflammatory mediators such as iNOS, IL-1β, and CCL2/MCP-1, while Dll4 suppression reduce expression of pro-inflammatory factors of so-called M1 macrophages (8, 39–41). More recently, we demonstrated that the interplay between ADP-rbosylation enzymes PARP9 and PARP14 regulates the balance of pro- vs. anti-inflammatory macrophages (36).

The loosely characterized anti-inflammatory or alternatively activated type of macrophages (traditionally called M2-like cells) is a general designation to refer to different subclasses that generate molecules that either suppress activity of the M1-like cells, initiate efferocytosis or promote resolution of inflammation (31) (Figure 1A). In mouse models, unlike in humans, these M2-like cells are identified with markers including arginase 1, mannose receptor C type 1, Ym1 and Fizz1 (38, 42). Although these markers do not identify M2-like macrophages in humans, information gleaned from using these murine M2-like cells may yield genes and proteins that are present and crucial in human macrophages for controlling inflammation. To produce M2-like phenotype *in vitro*, resting or activated macrophages need to be stimulated with any of the following chemokines: IL-4, IL-13, and IL-10, (42, 43) in contrast to the LPS or IFNγ stimulated M1-like cells. To avoid confusion, recent guidelines for macrophage subclass nomenclature propose to call these *in vitro* stimulated cells M(LPS), M(IFNγ), M(IL-4) or M(IL-10) (32).

IL-4 polarization of macrophages involve the Krüppel-like factor-4 (KLF-4) pathway and the IL-4/STAT-6 pathway. Paucity of KLF-4 in macrophages produce elevated expression of pro-inflammatory genes iNOS, and TNFα (44). MCP-1-induced protein (MCPIP) generated by KLF4 inhibits M1 activation. Likewise, it also promotes an M2-like phenotype. Furthermore, IL-4 triggered STAT6 induction promotes KLF4 expression, which mediates M2 activation through MCPIP activity as stated above (45). Peroxisome proliferator-activated receptors (PPARs) are also considered to be promoters of an anti-inflammatory phenotype (46). IL-4 and STAT-6 mediate transcription of several metabolism-related genes and regulators including PPARγ (47). Furthermore, absence of PPARγ in macrophages fail to induce oxidative metabolism and are also unable to exhibit M2-like phenotype (48). Independent from IL-4, IL-10 has recently been identified to promote mitochondrial fitness during pro-inflammatory activation of macrophages by mitigating the mitochondrial damage caused by reactive oxygen species (ROS) after iNOS activation, essentially paving the way for control, and resolution of inflammation (43).

Position along the macrophage phenotype/activation spectrum may simply be a function of the balance or imbalance of pro-inflammatory vs. anti-inflammatory factors present in every macrophage. Macrophages modeled *in vitro* are inherently heterogenous (Figure 1B). This means that although most of every single macrophage may be sitting at or near the extremes of the M1/M2 spectrum, some will remain near or at the median of the spectrum. The state of activation for groups of macrophages accumulating in local areas of the chronically inflamed tissue may be a function of the combined effect of the activation states of each macrophage single cell state. It is tempting to simplify that attenuating any and all M1-like macrophage populations will attenuate inflammatory burden. Disease state can therefore be reverted back to homeostasis. However, reducing pro-inflammatory activation without balancing the pro-resolving macrophage population and/or leaving a “patrolling” subpopulation might foreseeably lead to a more vulnerable immune system (8). That is why aggressive anti-inflammatory therapy targeting only macrophage accumulation and activation may increase incidence of severe infections. Calibrating the overall balance may lead to the development of efficient and safe therapies with minimal risk for unfavorable immunologic consequences. For example, inhibiting a specific signaling mechanism such as the Dll4-Notch pathway may suppress damaging macrophage products while promoting protective factors (39–41, 49).

### Mechanisms through metabolic reprogramming

#### Glycolytic energy preference of the activated macrophage

Evidence suggests that metabolic pathways are vital regulators of macrophage activation (50). As early as 1963, researchers recognized how metabolism affects monocyte and macrophage physiology. Based on experimental findings, alveolar macrophages, and circulating monocytes tend to have better physiologic function when energy is being fueled by aerobic respiration. Moreover, the macrophages were noted as capable of aerobic respiration in greater magnitudes than neutrophils and monocytes, and that inhibiting part of oxidative phosphorylation (OXPHOS) had a depressive impact on phagocytic activity (51). Later, G.C Hard showed that peritoneal macrophages from immune mice (activated) challenged by injecting a virulent strain of *C. ovis* intraperitoneally, had higher production of lactic acid compared to peritoneal macrophages from non-immune mice (resting macrophages). It was not clearly distinguished whether the peritoneal macrophages from the non-immune mice were truly differentiated and not an admixture of macrophages and patrolling monocytes in the peritoneal cavity. Still, his findings are among the first to show enhanced glycolysis through increases in lactate production along with decreased O_2_ uptake in immune activated primary macrophages (52). After more than a decade, experiments with thioglycolate-elicited mouse peritoneal macrophages show surprisingly high amounts of hexokinase, glucose-6-phosphate dehydrogenase and 6-phosphogluconate dehydrogenase implying further glycolytic induction in activated macrophages. In contrast, resting macrophages from saline injected mice showed less than 10% activity of 6-phosphofructokinase, complementing the prior observation that glycolytic rate is increased dramatically during phagocytosis or increased secretory activity (53). These early studies into macrophage metabolism research have broaden an entire field for mining biological mechanisms that allow us to appreciate immune activation as a reflection of metabolic state. Pivotal insights on macrophage metabolic reprograming came when it was reported that pro-inflammatory type polarization of macrophages with either LPS or INFγ could switch bioenergetic preferences of macrophages from OXPHOS to the glycolytic route (54, 55), a phenomena many observed to be reminiscent of the Warburg phenomenon happening in cancer cells (56–58). Other evidence supporting this, demonstrates that stimulation of TLRs by LPS increase *PFKFB3* (6-Phosphofructo-2-Kinase/Fructose-2,6-Biphosphatase 3) expression, resulting in an increase of the key glycolytic allosteric regulator fructose 2,6-bisphosphate and a glycolytic flux (59).

### Role of metabolites in classical activation of macrophages

Afterwards, work done in the lab of Luke O'Neill showed how metabolite key players in the glycolytic pathway, tri-carboxylic acid cycle (TCA) and OXPHOS play important roles in determining inflammatory activation states of immune cells like macrophages (56). They confirm that LPS causes macrophages to switch their core metabolism from OXPHOS to glycolysis. Inhibiting glycolysis could also suppress LPS mediated IL-1β secretion, albeit not TNFα, in mouse macrophages. While LPS does decrease expression of mitochondrial genes, there is a concomitant increase on levels of the TCA intermediate succinate. Their findings show that succinate: (1) stabilizes HIF-1α which support IL-1β release; (2) increases succinylation of several proteins; and (3) reduces desuccinylation of Sirt5 (60), a known epigenetic regulator of metabolism and inflammation. This Warburg-like phenomenon in macrophages implicate reprogramming at the epigenetic level through changing levels of acetyl-CoA potentially affecting increased acetylation and decreased deacetylations of histone proteins (61) Pro-inflammatory macrophages also have increased glucose transporter 1 (GLUT1) expression and availability to drive glucose uptake. This causes both hyper inflammatory proteome and transcriptome seen in RAW264.7 cells overexpressing GLUT1, leading to elevated secretion of inflammatory mediators, increase in reactive oxygen species (ROS) production and oxidative stress intracellularly (62) LPS-stimulated TLR4 increases mammalian target of rapamycin (mTOR) signaling, which induces the expression of lactate dehydrogenase and hypoxia induced genes (63, 64). Both events shunt acetyl-CoA away from TCA consumption. Moreover, this activation also leads up to elevated levels of an isoform of *PFKFB3* favoring glycolysis (63–65).

### Metabolic changes in anti-inflammatory type of macrophages

On the other hand, anti-inflammatory conditioning of macrophages have an opposite effect of promoting OXPHOS while reducing preference for glycolysis (66). IL-4 for instance promotes OXPHOS resulting in elevated oxygen consumption rate (OCR) in RAW264.7 cells. Extracellular acidification rate (ECAR), a measure of lactate production (anaerobic glycolysis) is also lower compared to pro-inflammatory LPS stimulated RAW264.7 cells (67). But as a caveat, LPS and/or IFNγ-polarized macrophages may not repolarize into an anti-inflammatory phenotype (IL-4 re-stimulation) if there is too much mitochondrial dysfunction brought out by LPS-induced hyper-glycolysis and nitric oxide production (68). This results in inhibition of OXPHOS in the mitochondria (66). This also prevents plasticity of LPS+IFNγ conditioned macrophages to convert into an anti-inflammatory phenotype (66) underlying the importance of OXPHOS in the resolution of inflammation or the direction of macrophage activation. In this case of proinflammatory macrophages resistant to phenotype switching, IL-10 may be the key signaling molecule that may aid IL-4 and accomplish the phenotype switch.

Arguably considered an anti-inflammatory chemokine, IL-10 could increase clearance of dysfunctional mitochondria through mitophagy. IL-10 activates STAT3 signaling which in turn activates DDIT4 transcription factor leading to inhibition of the NLRP3 inflammasome activated mTORC1 pathway, thereby releasing the restraint against autophagy. DDIT4 itself promotes clearance of ROS-damaged mitochondria by mitophagy while IL-10 maintains membrane gradient potential of mitochondria of primary macrophage promoting organelle integrity and fitness during macrophage activation. This allows for rapid resolution of inflammation (43). All these changes in bioenergetics during macrophage polarization may be accompanied by a changing metabolome and specifically, a shift in the lipidome as well.

### Lipidome changes in proinflammatory macrophages and the pro-resolving factors

E. Dennis' group did important work on macrophage lipidomics having contributed to the LIPID MAPS consortium to develop quantitative methods for evaluating the composition, biosynthesis, and function of all macrophage lipids. In one of their findings, they identified endotoxin Kdo_2_-Lipid A (KLA, a defined form of LPS) of *E.coli* activates macrophages via TLR4 similar to “regular” LPS. Prostaglandin D2 (PGD2) is the predominant eicosanoid produced after KLA—proinflammatory stimulation of RAW264.7 cells (> 120 ng/10^6^ cells), with almost nil 11-HETE production in contrast. PGE2 and PGD2 increase in a dose dependent manner with both LPS and KLA stimulation. PGE2 and PGD2 increase in a more potent but similar fashion to TNFα chemokine after LPS or KLA stimulation (69). Using RAW264.7 cells, their laboratory again assessed mouse macrophage lipidome changes with KLA treatment vs. drug perturbations like statins which are clinically relevant as statins inhibit cholesterol biosynthesis pathway and are able to reduce further inflammation in CAD of patients with high LDL profile (70). KLA tended to increase almost all categories of sphingolipid analyzed, cholesterol esters, and some glycrophospholipids. Statins like mevastatin (or compactin), a parent compound of pravastatin promote lipid changes intracellularly in these macrophages. Expectedly, statins blocked KLA induced increases in desmosterol and other components of the sterol biosynthetic pathway yet had no effect on actual intracellular cholesterol levels *per se* (71).

Still, there is more to uncover regarding metabolome and lipidome changes during the phenotypic differentiation by activated macrophages. With large metabolomic/lipidomic datasets, a systems approach to analysis may be the key to uncover comprehensive understanding of the dynamics of lipid and non-lipid metabolite pathways in the macrophage. This initial characterization in the mouse system is a first step to finding desirable therapeutic targets (71). However, there is still need to further elevate these studies to clinical relevance by using human samples. To this end, Tabas et al. further advanced the utility of mass-spectrometry lipidomic profiling (6) for atherosclerosis therapeutics by demonstrating specialized pro-resolving lipid mediators (SPMs) in atherosclerotic plaques. SPMs are generally essential fatty acids-derived autacoids (2) They found that bioactive lipid derivative resolvin D1 (RvD1) levels were low relative to pro-inflammatory lipid leukotriene B_4_ (LTB_4_) in vulnerable plaques of the human carotid artery. This was further confirmed in hyperlipidemic mouse models by showing that administration of RvD1 increases plaque stability, lowers oxidative stress and necrosis, and thickens fibrous caps. Their findings support a–omics assisted mechanistic rationale for SPM therapy in CAD to mitigate plaque vulnerability (37).

Another set of SPMs, the maresins which are studied extensively by the Serhan group, are macrophage derived and are produced via 14-lipoxygenation of docosahexaenoic acid (DHA) that is either converted by enzymes into mediators with two-OH groups or into autacoids that are peptide-lipid conjugates, called maresin conjugates. These SPMs promote the uptake and clearance of apoptotic cells by macrophages. Maresins also regulate portions of tissue repair (2) therefore, resolution of the inflammatory damage. SPMs can potentially influence switching of macrophage function.

### Macrophage crosstalk with other immune cells

Not only are macrophages known for their plasticity, these cells have the ability to influence and be influenced by other immune cells like T cells that results in a similar macrophage phenotype switching as above. The reciprocity of T cells and macrophages through either paracrine signaling or molecular interactions may dictate the direction of inflammation. It may either drive well into a vicious cycle of unmitigated chronic pro-inflammatory atherosclerotic events or toward inflammation resolution (72). Several reports have already shown that these macrophages communicate with other immune cells via specified protein pairs on their cell surfaces. Pairs can be co-stimulatory or co-inhibitory, whereby molecular interaction between these pairs triggers downstream biomolecular cascades that may promote or limit macrophage activation in atherosclerosis (73). Oncology researchers took advantage of this T cell-macrophage crosstalk, in order to combat cancer cells (74). Immunologists have coined the term immune checkpoints to identify these pairs of proteins that interact to either promote a pro-inflammatory activation or an anti-inflammatory one (75).

One of these pairs of co-stimulatory proteins are CD40 and CD40L (ligand) which are expressed in macrophages among other cell types found in atherosclerotic vessels (76). Both CD40 and CD40L are expressed highly in atherosclerotic lesions (77) and plasma which may predict patients with features of high-risk atherosclerotic lesions corroborated with MRI (78). Abrogating CD40L activity effectively reduces release of pro-inflammatory factors together with reducing activation of macrophages by activated T cells *in vivo* using mouse models of atherosclerosis. Deletion of either CD40 or CD40L has atheroprotective effects by mitigating macrophage activation (73). CD80/B7-1 and CD86/B7-2 are a pair of recognized M1-like markers for macrophages are also expressed in dendritic cells (DCs) in atherosclerotic plaques. They co-stimulate and bind to CD28 on T cells, B cells, and other macrophages (79). CD80 and CD86 double deficiency in hyperlipidemic LDL receptor-deficient (LDLr^−/−^) mice results in lesser atherosclerotic burden (80) Other co-stimulatory immune checkpoint proteins found in human atherosclerotic lesions and in pre-clinical models that tend to skew macrophages and DCs to the pro-inflammatory end of the spectrum are: OX40-OX40L, CD137-CD137L, and CD30-CD30L (81) Immune checkpoint and co-inhibitory proteins PD-1 and PD-L1/2 inhibit T cell immune response resulting in a beneficial atheroprotective effect. Stimulation of PD-L1 expression *in vitro* could attenuate the stimulatory ability on allogeneic T cell proliferation and its cytokine production, including IFNγ (82). Such effect, however, is detrimental to immune clearance of tumor cells in cancer, hence the success of the anti-PD-1 immunotherapy oncologic drug pembrolizumab (83). CD27-CD70 is a another possible co-inhibitory pair since CD27 paucity on mice models show markedly increased atherosclerotic burden. In addition, CD27 is demonstrated to be essential in maintain a healthy pool of regulatory T cells (Tregs), preventing increased apoptosis of Tregs (84). CD70 promotes macrophage function and viability, and is important for effective efferocytosis and extrusion of oxLDL. CD70 deficiency results in more advanced atheroma (85). These are just a few of the immune checkpoints that affect macrophage behavior. Other proteins include, though not limited to, CTLA-4, ICOS-ICOSL, GITR-GITRL, and TIM (81). Other potential macrophage checkpoints are CD47 and signal regulatory protein alpha, SIRPα. In cancer, activation of SIRPα by CD47 on macrophages suppress both phagocytosis and respiratory burst (74). Therefore, as expected in mouse atherosclerotic models, blockade of CD47 exerts anti-atherosclerotic effects, halting lesion progression and preventing plaque rupture and restores phagocytosis/efferocytosis (86). A more recently studied immune regulator is the V-domain containing Ig Suppressor of T cell Activation (VISTA, aka PD-1H, DD1α; gene name DIES1). VISTA is both a receptor and a ligand with immunosuppressive effects on IFNγ, and TNFα with T cells and macrophages (74). In fact, majority of VISTA+ macrophages have the anti-inflammatory M2-like phenotype (87). Other proteins that can potentially act as immune checkpoints by control macrophage behavior via T cell and other immune cell 2-way interaction include: T cell immunoglobulin and ITIM domain (TIGIT) and indoleamine-2,3-dioxygenase (IDO). More studies are, however, required to clarify their roles in the macrophage inflammatory phenotype spectrum (74).

A summary comparison of these biomolecular signatures of atherosclerosis, as evidenced by findings in studies of mouse vs. human macrophages is enumerated in Table 1.

**Table 1:** Comparison of the effects of some biomolecular markers of macrophage activation as seen in mouse *in vitro* & pre-clinical models of atherosclerosis vs. human atherosclerosis (including *in vitro* studies).

**Biomolecular markers of macrophage activation**	**Effect on pro-inflammatory activation of macrophages(+/- mouse models of atherosclerosis)**	**References**	**Effect on pro-inflammatory activation of macrophages/expression in plaques of clinical atherosclerosis**	**References**
NLRP3 inflammasome Cholesterol crystals	↑	(20)	↑	(88)
IL-1β	↑	(20, 89)	↑	(4)
IL-6, TNF-α	↑	(38)	↑	(90, 91)
TLR2/TLR4	↑	(92, 93)	↑	(94)
iNOS	↑	(95)	↑	(96, 97)
INFγ/STAT1 signaling	↑	(36)	↑	(98)
CCL2/MCP1	↑	(38)	↑	(99, 100)
IL-4, IL-13 signaling	↓	(42)	?	(101)
IL-10 signaling	↓	(43)	↓	(102)
Dll4/Notch1 signaling	↑	(38, 41, 103)	↑	(39)
Ym1	↓	(104)	*No correlation*	(105)
Fizz1	↓	(104)	↑ ?	(106) (107)
PPAR-α/-γ	↓	(46)	↓ (predicted for PPAR-α)?	(108)
CD40 –CD40L	↑	(109)	↑	(76)
CD80-CD86	↑	(80)	↑ (*in vitro*, DC only)	(110)
OX40-OX40L	↑	(103)		(111)
CD137-CD137L	↑	(112)	↑ (monocytes)	(113)
CD30-CD30L	↑ (CD30L only in LPS-RAW264.7 cells)	(114)	↑ (CD30)	(81, 115)
PD1-PD-L1/2	↓ by PD1 (Chen)/ ↑ by PD-L1/2 (Gotsman)	(116) (117)	↓ (myeloid DC)	(82)
CD27-CD70	↓ (CD70)	(84)	*?*	No definitive consensus
Hyperglycolysis	↑	(56)	↑	(118)
GLUT1	↑	(119)	↑ (*in vitro* only)	(120)
OXPHOS	↓	(56)	*?*	*No evidence*
Citrate	↑	(56)	*?*	*No evidence*
Succinate	↑	(60)	*?*	*No evidence*
Itaconate	↑	(56)	*?*	*No evidence*
Prostaglandin D2	↑	(69)	↑	(121)
Prostaglandin E2	↑	(69)	↑	(122)
Resolvins	↓	(1, 37, 123)	↓	(124)
Maresins	↓	(123)	↓	(125)

*DC, dendritic cells; OXPHOS, oxidative phosphorylation pathway*.

### Macrophage heterogeneity in atherosclerosis

Advances in single cell analysis provided insights of how heterogenous human primary macrophages are. Single cell qPCR reveals how heterogenous resting macrophages, M0 or M(-), are. Yet after a pro-inflammatory polarization by IFNγ activation, M(IFNγ) macrophages even have a further heterogenous response showing subpopulations that are more responsive to pro-inflammatory signals, even as some populations remain resistant and reticent, maintaining an unstimulated phenotype (35, 36). As cited earlier, identifying these macrophages and contrasting them vs. the highly responsive ones may help filter out by enrichment of key regulatory genes. These genes may promote phenotype switching thereby unraveling them to be desirable therapeutic targets. These targets or pathways may be otherwise hidden when examining an average transcriptomic readout from “bulk” macrophage populations processed from conventional qPCR or mRNAseq assays instead of the high resolution readouts of single cell analysis.

## Future perspectives: new paradigms of discovery science and drug development in vascular inflammation

**Figure 2:**
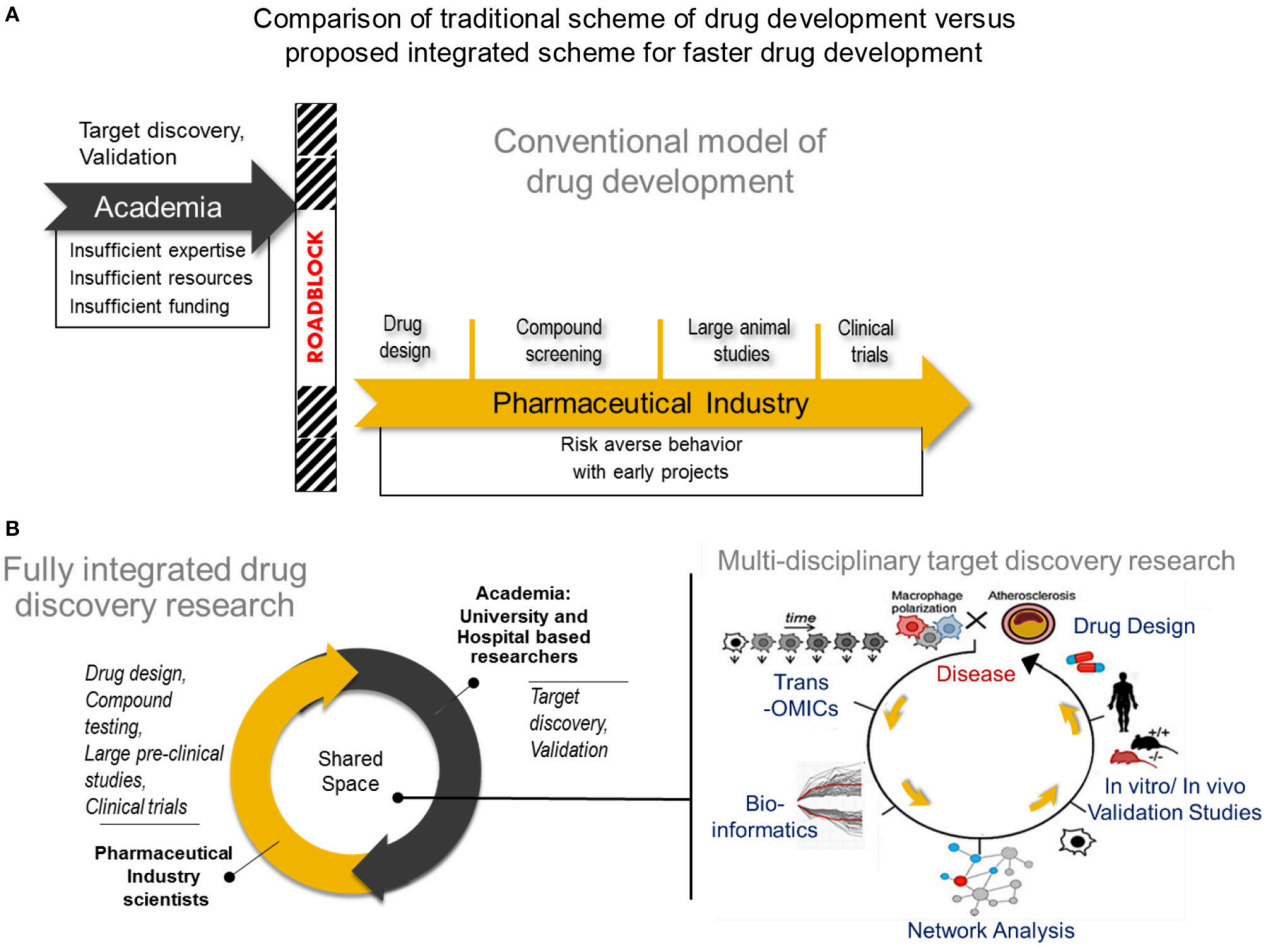
Comparison of tradional drug development vs. proposed integrated drug discovery research **(A)** The conventional model of drug development Target discovery and validation studies are often done by academic researchers who usually have insufficient expertise and resources to conduct such studies resulting years of development. After characterizing a potential drug target, many academic investigators struggle to translate their finding into the pharmaceutical space due to a natural disconnect between academia and industry. In rare instances, academic investigators may manage to cross the roadblock and transfer their breakthroughs to industry, followed by a lengthy process of drug design, compound testing and animal studies before the drug will be considered for human studies. **(B)** A new paradigm for drug development Center) A fully integrated drug discovery research in our laboratory involves close collaboration between academic and pharmaceutical industry scientists. Right) Use of multi-omics approach to disease characterization with systems approach analysis for faster target discovery and prioritization and drug design. A right panel was reproduced from Iwata et al. (36). Trans-OMICs: genomics, transcriptomics, proteomics, epigenomics, metabolomics, lipidomics, etc.

With the multitude of mechanistic perspectives that make up the macrophage behavior in atherosclerosis, there are many aspects to consider when designing effective therapies with the highest potential of leaping from the laboratory bench to clinical translation. Consolidating these various angles intelligently, in order to arrive at a viable drug target in the fastest possible way, is an attractive goal for the medical science community. However, due to limited resources, insufficient funding, and lack of expertise, many academic investigators may fail to develop and deliver their target discovery beyond the laboratory space (126, 127). Despite the evident strengths of pharmaceutical industry in drug development, its recent tendency to avoid investing in early, high-risk projects appears to have enhanced this gap in translation of academic target discovery into the clinic (128, 129). These roadblocks often pre-maturely remove the possibility of an otherwise promising target from being transferred to pharmaceutical development and eventually becoming a novel, even first-in-class, drug. (Figure 2A). Arriving at the most cost effective strategy for pharmaceutical translation requires a balancing act of carefully prioritizing the targets discovered with very rational and discreet management of resources for target(s) validation while still within the hands of the academic researcher (130).

Close collaboration between academia and industry will foster faster exchange of expertise and resources that could lead to mutually beneficial research outputs (130, 131). In such a model established in our laboratory (Figure 2B, center panel), drug development is facilitated when effectively combining the exploratory nature of innovative academic research and the extensive expertise in drug design and the rigor and resource intensive validation from pharmaceutical industry. Much advancement in computational biology and networks medicine, as well as steadily declining costs and time required for—omics experiments and single cell experiments also help to speed the transition of academic target discovery into drug development. It is easier now to consolidate various biological mechanisms that define macrophage activation, and use an intergrated approach to arrive at viable targets in a more comprehensive and unbiased manner. A typical workflow of target discovery research in our laboratory is demonstrated in Figure 2B (**right panel**). We expect that paired with an ever-expanding knowledge base and expertise in performing these–omics experiments, as well as use of machine learning approaches, a more solid understanding of pathways that drive macrophage activation will emerge. Novel pathways of activation may be uncovered when we actively use network science to incorporate comprehensive readouts from–omics and single cell experiments. With all these innovative large-scale approaches in biological research and data analyses, pairing with synergistic efforts from academic and industry scientists and cell and computational biologists, more effective and better defined drugs may arrive to the market sooner. Better understanding of biological mechanisms for macrophage activation and heterogeneity through big data, particularly of clinical samples, and integrated analysis, may also lead to safer drugs that target specific subsets of populations as opposed to a more generalized approach.

## Author contributions

JD and MA both contributed to the concept and key contents of this manuscript. JD drafted the manuscript and MA critically reviewed and edited it.

### Conflict of interest statement

The authors declare that the research was conducted in the absence of any commercial or financial relationships that could be construed as a potential conflict of interest.
